# The Effects of Physical Activity on Health and Quality of Life in Adolescent Cancer Survivors: A Systematic Review

**DOI:** 10.2196/cancer.5431

**Published:** 2016-05-24

**Authors:** Amanda Wurz, Jennifer Brunet

**Affiliations:** ^1^ School of Human Kinetics Faculty of Health Sciences University of Ottawa Ottawa, ON Canada

**Keywords:** exercise, controlled clinical trial, randomized controlled trial, review, treatment effectiveness

## Abstract

**Background:**

There are numerous published controlled trials assessing the safety and the benefits of physical activity (PA) for child and adult cancer survivors. However, trials exclusively comprised of adolescent cancer survivors aged 13-19 years, who may experience different health and quality of life (QOL) effects as a function of their developmental status, are lacking. Rather, some trials have included both adolescent and child cancer survivors together.

**Objective:**

The aim of this systematic review was to synthesize the findings from randomized controlled trails (RCTs) and controlled clinical trials (CCTs) investigating the effects of PA on health and QOL outcomes in samples comprised of >50% adolescent cancer survivors to summarize the current state of evidence, identify knowledge gaps, and highlight areas in need of additional research within this population.

**Methods:**

Using a search strategy developed for this review, 10 electronic databases were searched for RCTs and CCTs that reported on the effects of PA on at least 1 health and/or QOL outcome in samples comprised of >50% adolescent cancer survivors.

**Results:**

From the 2249 articles identified, 2 CCTs met the predetermined eligibility criteria and were included in this review. Combined, 28 adolescents (of 41 participants) who were receiving active treatment participated in the 2 studies reviewed. A total of 4 health and QOL outcomes (ie, bone mass, fatigue, grip strength, QOL) were assessed pre- and post-PA intervention.

**Conclusions:**

On the basis of the 2 studies reviewed, PA appears to be safe and feasible. PA also shows promise to mitigate reductions in bone mass and might be a viable strategy to improve fatigue, grip strength, and QOL. High-quality controlled trials with larger samples exclusively comprised of adolescent cancer survivors that assess a wide range of outcomes are needed to determine the effects of PA on health and QOL outcomes in this population.

## Introduction

Each year in North America, more than 7500 adolescents are diagnosed with cancer [1,2], thereby becoming a “cancer survivor” as the National Cancer Institute defines a cancer survivor from the point of diagnosis onward [3]. Accordingly, a cancer survivor is a person who may be awaiting treatment, be actively receiving treatment (ie, on-treatment), or have completed treatment (ie, off-treatment). Approximately 80% of adolescents will live at least 5 years after they are diagnosed with cancer [4]. Although this rate reflects a relatively good prognosis, adolescent cancer survivors often report negative side effects (eg, fatigue, pain [5]) and have an increased risk of disability, morbidity, and premature mortality [6-10]. Furthermore, they may have impaired psychological and social functioning [11,12], which can hinder their health and quality of life (QOL [13,14]).

There have been several trials testing the effects of physical activity (PA) on health and QOL outcomes among cancer survivors [eg, 15-18], and several reviews have summarized the findings [eg, 19-22]. Combined, this work shows that PA can help to reduce the risk of disability (eg, physical limitations, neurocognitive impairments), morbidity (eg, obesity, diabetes, cardiovascular diseases, second cancers, organ dysfunctions), and premature mortality. This work also shows show that PA may decrease some of the negative side effects reported by cancer survivors, as well as improve health and QOL across different domains of functioning (ie, physical, psychological, emotional, social [15-22]). In recognition of these benefits, several cancer organizations recommend that cancer survivors incorporate PA into their daily lives [23,24], and many groups have developed PA guidelines for cancer survivors [25,26]. Notwithstanding the contributions of existing trials and reviews, none have focused exclusively on adolescent cancer survivors [15-22], preventing the development of age-appropriate PA guidelines for this population.

The Public Health Agency of Canada defines adolescents as individuals aged 13-19 years [27]. This life stage is characterized by the onset of puberty, a period when a number of biological, physical, psychological, emotional, social, and cognitive changes occur [28,29]. A diagnosis of cancer during this time may cause deviations from normative developmental changes [30]. For example, chemotherapy and radiation can result in precocious puberty, gonadal dysfunction, and infertility [31,32]. These treatments may also result in growth hormone deficiency, which has been related to decreased muscle mass, reduced PA tolerance, and impaired growth [33]. Furthermore, some of the physical challenges associated with cancer and its treatments markedly reduce PA levels [34], which may partly explain the increased odds of adolescent cancer survivors being obese by 2.59 for females and 1.86 for males compared to their siblings without a history of cancer [35]. These biological and physical challenges and changes can negatively impact adolescents’ body image and adversely affect their physical, psychological, emotional, and social functioning [36]. Thus, cancer and its treatments may affect adolescents’ psychological, social, and cognitive development, which can impair their ability to master necessary skills in these areas [37-39]. It can also lead to psychological, social, and cognitive maladjustment such as anxiety, depression, poor social relations, and lower educational and/or occupational attainment [37-39]. It is therefore imperative to determine if PA can be used as a strategy to reduce the negative effects of cancer on adolescents’ normative biological, physical, psychological, emotional, social, and cognitive development.

Given that adolescence is a time of tremendous growth and development [28,29], which can be challenged by cancer, the findings from PA trials and reviews focused on younger and older cancer survivors should be extrapolated cautiously and efforts should be made to examine the effects of PA on these outcomes in adolescent cancer survivors specifically. Hence, the aim of this systematic review of the literature was to synthesize the findings from randomized controlled trails (RCTs) and controlled clinical trials (CCTs) investigating the effects of PA on health and QOL outcomes in adolescent cancer survivors to summarize the current state of evidence in this population, identify knowledge gaps, and highlight areas in need of additional research.

## Methods

The review was carried out following established criteria for the good conduct and reporting of systematic reviews (ie, Preferred Reporting Items for Systematic Review and Meta-Analyses, Cochrane Handbook for Systematic Reviews of Interventions, Consolidated Standards of Reporting Trials, Guidance on the Conduct of Narrative Synthesis in Systematic Reviews [40-45]). The full review protocol is published elsewhere [46].

### Search Strategy

First, 10 electronic databases were searched (ie, CINAHL, Cochrane Central Register of Controlled Trials, EMBASE, LILACS, MEDLINE, PEDro, Physical Education Index, PsycINFO, PubMed, SPORTDiscus) for articles published in English in peer-reviewed scientific journals from database inception to November 2015. A combination of Medical Subject Heading terms and keywords covering the target population (eg, adolescent, young person, teen, cancer patient), intervention (eg, exercise, PA, physical fitness), and comparison condition (eg, control group, usual care) were used after consultation with an expert librarian (YL). Of note, these search terms were revised and refined after conducting an initial search. The rationale for this is presented in the published systematic review protocol [46], along with additional details on the specific search strategy and how it was developed. Next, the reference lists of all relevant articles identified in the electronic databases were searched to identify additional studies.

### Selection of Studies

Both authors screened the titles and abstracts of all studies identified during the search using the following predetermined eligibility criteria: (1) reported the effect(s) of PA on at least 1 health and/or QOL outcome, (2) used a RCT or CCT study design, (3) had at least pre- and post-intervention assessments, and (4) had a sample comprised of >50% cancer survivors aged 13-19 years. The latter criterion was based on precursory knowledge that no PA trials have been conducted with samples exclusively comprised of adolescent cancer survivors. For the purpose of this review, an intervention was considered as anything greater than 1 PA session. Studies were excluded if they had multiple program features that could be attributed to the outcomes reported or if they had insufficient details on the target population, intervention, comparison condition, and/or outcomes (after the study authors were contacted by email and it was determined that the requested data were unavailable). The inter-rater agreement between both authors on the eligibility of studies was >95%, representing a high level of agreement [47]. In instances of disagreement during the review process, consensus was reached through discussion with 2 independent researchers (AJ and CO).

### Data Extraction

Both authors extracted the following information from the eligible articles: (1) study characteristics (ie, year of publication, country, study design), (2) sample characteristics (ie, number of participants randomized, age, type(s) of cancer diagnosed, treatment status), (3) intervention characteristics (ie, supervision, setting, length, frequency, duration, intensity, activity types(s)), (4) outcome measures, and (5) outcomes (ie, health, QOL). Additional relevant information such as the use of theory and whether intention-to-treat analysis was performed were also recorded. In cases where details were missing, authors were contacted by email*.*

## Results

**Figure 1:**
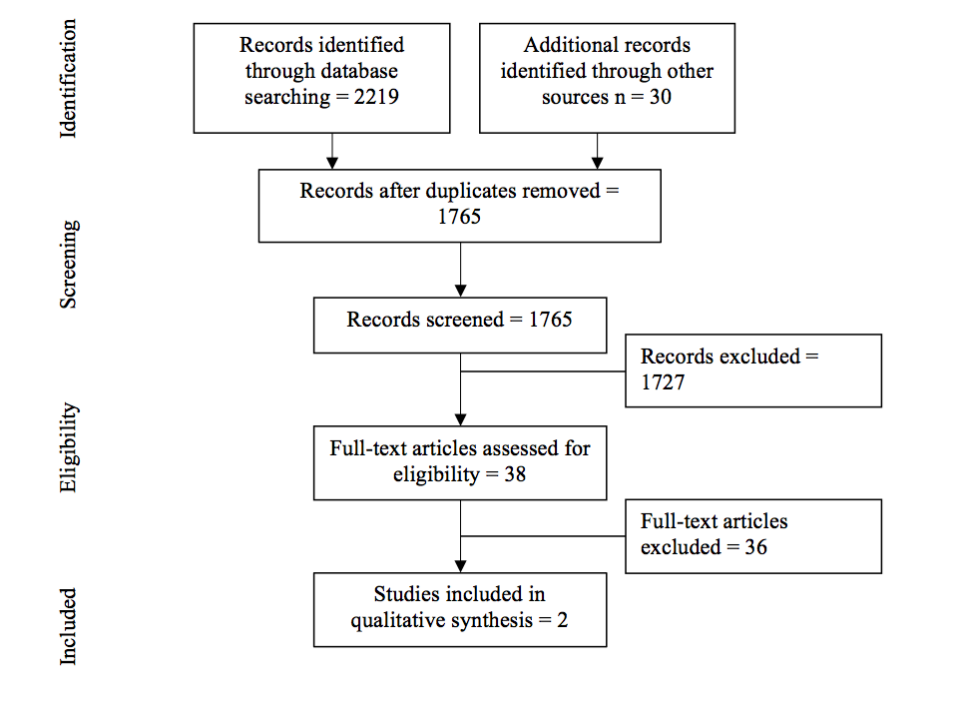
Flow chart of search results.

As illustrated in Figure 1, a total of 2249 articles were identified during the search, of which 2219 were from the electronic search and 30 were from the manual screening of reference lists. After 484 duplicates and 1727 articles that did not meet eligibility criteria were excluded, 38 full-text articles were considered potentially relevant. Both authors independently reviewed the full-text articles and determined that 36 studies were not eligible for review for the following reasons: study was not published in English (n=1), no full-text was available (n=12), no control group (n=3), no PA intervention (n=6), multiple program features (n=1), protocol article (n=2), no health and/or QOL outcome reported (n=1), and age criteria not met (n=10). For those studies that were excluded because of the age criteria, participants were either above the defined age range [48-55] or samples were comprised of ≤50% adolescents [56,57]. This left 2 articles that met the predetermined eligibility criteria [58,59].

###  Study Characteristics

Multimedia Appendix 1 provides an overview of the characteristics of the reviewed studies. Müller et al [58] published their CCT in 2014 and Rosenhagen et al [59] published their CCT in 2011. Both studies were conducted in Germany [58,59].

### Sample Characteristics

There were a total of 41 participants between the 2 studies. Müller et al [58] included 21 participants (mean age = 14.0 years; 67% (ie, 14 of 21) were aged 13-19 years), who were diagnosed with malignant bone tumors in the lower extremity and were currently receiving adjuvant treatment. Rosenhagen et al [59] included 20 participants (mean age = 15.3 years; 70% (ie, 14 of 20) were aged 13-19 years), diagnosed with mixed cancer during the isolation phase of peripheral blood stem cell transplant.

### Intervention Characteristics

Both interventions varied considerably with regard to the type of PA intervention and reported characteristics. Thus, each intervention is described separately. Müller et al [58] delivered an in-hospital PA intervention during participants’ inpatient stay (range = 8-12 inpatient stays) over a 6-month period. PA sessions were offered from Monday to Friday. Participants who were not able to leave the ward to attend the session had the opportunity to complete the PA session in their hospital room. Participants were advised to attend the PA sessions at least every second day while admitted as an inpatient. Sessions lasted 15-45 minutes and included aerobic activity (ie, stationary bicycling, walking or jogging on the treadmill, using an elliptical trainer), strength training (ie, multiple joint exercises such as squats, lunges, rowing), balance and flexibility training, and sports games (eg, football, basketball, table tennis) at moderate-to-vigorous intensity. Borg’s Rating of Perceived Exertion Scale was used to monitor PA intensity. All sessions were supervised by 2 trained sports therapists [58].

Rosenhagen et al [59] delivered an in-hospital PA intervention during participants’ inpatient stay over a 5- to 7-week period. PA sessions were offered 3 times/week in participants’ hospital room. Sessions lasted 50 minutes and included moderate-intensity aerobic activity (ie, stationary bicycling) and strength training using barbells, balls, and participants’ own body weight (eg, squats, side steps, balancing on 1 leg). A heart rate monitor was used to ensure participants engaged in PA at the intended intensity. Trained sports therapists supervised each session [59].

### Intervention Outcomes

The results of the PA interventions are presented in Multimedia Appendix 2. The 4 health and QOL outcomes that were assessed included: (1) bone mass, (2) fatigue, (3) grip strength, and (4) QOL. PA levels, intervention acceptance, intervention adherence, and adverse events were the non-health or QOL outcomes that were reported.

#### Health and QOL Outcomes

Müller et al [58] assessed bone mass with dual-energy X-ray absorptiometry, Lunar Prodigy System (enCore 2006, Software version 10.51.006; GE Healthcare, Madison, WI, USA). Changes analyzed using multivariate analysis of covariance showed decreases in bone mineral content (BMC), bone mineral density (BMD), and height-corrected lumbar spine *Z*-scores over the course of the intervention and at follow-up in both groups. Despite decreases in BMC, BMD, and height-corrected *Z*-scores in both groups, these declines were attenuated (nonsignificantly) in the intervention group compared with the control group. There was no significant difference between groups in BMC over the course of the intervention and at follow-up. There were significant differences in lumbar spine BMD and height-corrected lumbar spine *Z*-scores in the intervention group compared with the control group post-intervention; however, this difference was no longer significant at follow-up [58].

Rosenhagen et al [59] assessed fatigue and found that participants’ symptoms of fatigue improved, albeit not statistically significantly, over the course of the study for those assigned to the PA intervention. It is not possible to determine if fatigue differed between the intervention and control groups as no comparisons between groups were made [59].

Rosenhagen et al [59] assessed grip strength using a hand-held dynamometer (JAMAR; Homecraft Ltd, Kirby-in-Ashfield, Nottinghamshire, UK). On average, grip strength increased nonsignificantly from baseline to day 14, but returned to baseline levels post-intervention. It is not possible to determine if grip strength differed between the intervention and control groups as no comparisons were made between groups [59].

Rosenhagen et al [59] assessed QOL using participant reports on the German version of the KINDL and its associated oncology subscale [59,60]. Over the course of the study, participants in the intervention group reported a nonsignificant U-shaped trend in general QOL, that is, levels decreased from baseline to day 14 and then increased from day 14 to post-intervention. Cancer-specific QOL increased nonsignificantly over time in the intervention group. It is not possible to determine if general or cancer-specific QOL differed between groups as no comparisons were made between groups [59].

#### Other Outcomes

Müller et al [58] assessed daily PA levels using accelerometers (StepWatch 3^TM^ Activity Monitor; Orthocare Innovations, Washington, DC, USA). Post-intervention (ie, 6 months after baseline) and follow-up (ie, 12 months after baseline) levels of PA were assessed in the intervention and control group. At post-intervention, the intervention group engaged in more PA (16.9 minutes/day) than the control group (1.7 minutes/day), and the effect size for this difference was large. Also, at follow-up, both the intervention and control group increased their levels of PA. However, the intervention group continued to engage in more PA (25.2 minutes/day) than the control group (8.0 minutes/day), and the effect size for this difference was large [58].

Rosenhagen et al [59] assessed participants’ acceptance of the intervention by asking participants to discuss their general opinion of the inpatient PA intervention during semi-structured interviews. For those assigned to the intervention group, they were asked to think about the PA intervention they participated in, whereas for those assigned to the control group, they were asked their opinion after receiving a description of the PA intervention delivered to those in the intervention group. Overall, participants in the intervention group held positive opinions, whereas the control group expressed skepticism about participating in such an intervention because of the additional burden they perceived it would have [59].

Müller et al [58] assessed adherence and operationalized it as the number of times the intervention was received (mean = 34.5 ± 8 PA sessions) out of the total amount of times the intervention was to be delivered (mean = 44.8 PA sessions). They reported an adherence rate of 77% [58]. Rosenhagen et al [59] did not report on adherence. No adverse events were reported in either of the PA interventions, leading the authors of the 2 studies included in this review to conclude that their PA interventions were safe [58,59].

## Discussion

### Principal Findings

This systematic review summarizes the best available evidence regarding the effects of PA on health and QOL outcomes for samples comprised of >50% adolescent cancer survivors. A total of 2 CCTs were identified that had mixed samples of children and adolescents [58,59]. Although there was a lack of statistical significance for most outcomes, trends in the data show that PA may be a useful strategy to improve health and QOL in adolescent cancer survivors. Specifically, the studies found that bone mass, fatigue, grip strength, and QOL were maintained or improved in the PA intervention group. Simple inspection of mean values demonstrated that PA may confer clinically meaningful changes (ie, experienced as relevant by the participants). Indeed, researchers have suggested that the smallest change in a treatment outcome that patients would identify as important signifies a clinically meaningful effect [61].

Given the evidence that PA can improve symptoms of fatigue in adult cancer survivors [62,63], Rosenhagen et al [59] tested whether a PA intervention could improve symptoms of fatigue in child and adolescent cancer survivors. Although fatigue scores improved over the course of the intervention, the change was not statistically significant. This is in contrast to the overwhelming evidence that PA does improve symptoms of fatigue in adult cancer survivors [62,63], and the emerging evidence with samples comprised of both children and adolescents. For example, Yeh et al [57] delivered a 6-week home-based PA intervention using active video games to a sample of child and adolescent cancer survivors (ie, 32% adolescents, 7 of 22 participants).Participants reported improved mean fatigue scores in the intervention group over the course of the intervention; however, these changes did not result in statistically significant differences between the intervention and control group when intention-to-treat analysis (which considers the outcomes of all participants regardless of whether they received their assigned treatment) was used [57]. In contrast, when per-protocol analysis (which considers only the outcomes of participants who received their assigned treatment) was used, general fatigue scores were significantly different between the intervention group and the control group at 1-month follow-up [57]. Given the divergent findings both across studies and within studies depending on the analytical approach, more research is necessary to determine the efficacy of PA to improve symptoms of fatigue in adolescent cancer survivors. Furthermore, researchers should explore how improvements in symptoms of fatigue may in turn promote other positive physical and psychosocial outcomes (eg, emotional well-being, social engagement, cognitive functioning) in adolescent cancer survivors.

Although the included studies had samples comprised of 67% [58] and 70% [59] adolescents, it must be underscored that no published controlled trials examining the effects of PA in a sample exclusively comprised of adolescent cancer survivors aged 13-19 years were identified when this review was conducted. Both studies reviewed had survivors as young as 6 and 8 years. This is an important consideration that should be taken into account when interpreting the findings from the included studies. Moving forward, researchers seeking to study the effects of PA on health and/or QOL outcomes in adolescent cancer survivors could conduct trials at multiple sites to enroll larger samples of adolescents. Researchers could also consider reporting results for adolescents separately in cases where they have mixed samples that include children and/or adult cancer survivors. For example, although not reviewed herein because adolescents comprised a minority of the sample (ie, 38% adolescents, 11 of 29 participants), Hinds et al [56] separated data for the adolescent cancer survivors in their enhanced-activity intervention. However, the authors only reported the *P*-values for the total sample in the Results section, thereby making it difficult to determine if there were significant differences in the outcomes of interest (ie, sleep efficiency, fatigue) for adolescents.

In line with a previous systematic review conducted with pediatric cancer survivors aged 2-21 years [19,20], the reviewed studies had small sample sizes [58,59]. It is therefore plausible that the authors of the included studies did not detect statistically significant effects because they lacked sufficient statistical power. It is also possible that the intensity and duration of PA was insufficient to produce change in the outcomes assessed. In addition, given the evidence that long-term involvement in PA may be needed to affect health (ie, fatigue, strength) and QOL outcomes [64-66], the interventions may not have been long enough to affect the studied outcomes. Accordingly, it is possible that increasing the dose of PA, and offering interventions lasting longer, may yield statistically and clinically significant effects on health and QOL. Both studies included reported on the effects of PA for adolescent cancer survivors who were undergoing treatment [58,59]. Considering that treatments are associated with severe declines in physical, psychological, emotional, social, and cognitive functioning [5-14], it is conceivable that small improvements, or even maintenance, may translate into clinically important differences in health-related outcomes for this population.

The effects of PA on a wider range of health and QOL outcomes cannot be established as the CCTs included in this review focused mainly on physical health outcomes (ie, bone mass, fatigue, grip strength [58,59]). Considering that adolescence is a time of tremendous biological, physical, psychological, emotional, social, and cognitive development [28,29], it is especially important to assess outcomes in each of these areas to determine if PA can help promote optimal development for adolescent cancer survivors. For instance, biological changes (eg, impaired growth, weight gain or loss, early or delayed sexual maturation [30-35]) may lead some adolescent cancer survivors to feel physically different from their peers, heightening their experiences of body dissatisfaction, which may manifest as social anxiety, psychological distress, and avoidance of health protecting behaviors such as PA [36,67]. In addition, psychological, social, and cognitive development (eg, establishing autonomy and independence, building social skills and coping resources) may be negatively impacted by cancer and its treatments [37-39]. It is therefore necessary to determine if PA can facilitate the development of positive health behaviors, improve body image, mitigate psychosocial maladjustment (eg, anxiety, psychological distress, depression, social skills/functioning), and address cognitive limitations (eg, fine motor, visual-spatial and nonverbal skills, attention, concentration).

Although for the most part, PA was not shown to significantly affect adolescent cancer survivors’ health and QOL at the conventional 5% level of significance, it is important to balance the lack of evidence based on the reviewed CCTs with the previously mentioned limitations of each. Combined with evidence from case-series studies linking PA to improvements on various outcomes in this population (eg, [68]) and the overwhelming evidence for the benefits of PA in pediatric and adult cancer survivors [19-22], it seems prudent to recommend that adolescent cancer survivors engage in PA, especially given the lack of adverse events in the reviewed studies. Indeed, based on previous reviews with pediatric [19,20] and adult cancer survivors [21,22], the low dropout rates, high adherence to the intervention protocols, and ability to recruit participants in the studies reviewed, it can be concluded that PA is not only safe but also feasible for adolescent cancer survivors undergoing treatment. As such, health care providers may recommend PA to their patients without fear of harm, provided they take into account contraindications that would make PA potentially inadvisable for certain patients (eg, cardiopulmonary disease, neurological problems, impairments in general performance limiting mobility [25,26]). To date, few resources exist for health care providers who want more information regarding the safety and benefits of PA for adolescent cancer survivors [26]. Thus, many groups are developing resources. For example, based on the findings of this review, and recommendations from other research and resources [15-26], the authors of this manuscript are developing a PA pamphlet that health care providers may give to adolescent cancer survivors. In the meantime, health care providers can encourage adolescent cancer survivors to engage in PA. Health care providers should also take into account adolescents’ past PA behavior, current physical condition, contraindications, and PA preferences and may also consider referring adolescent cancer survivors to a PA specialist who has received training in cancer and PA.

### Limitations

The limitations of the current review should be taken into account. First, although the strength of conducting a systematic review is the ability to integrate and pool existing data to draw firm conclusions and determine effect sizes [69], the lack of studies and the variability in the interventions and outcomes reported in the studies reviewed prevented this. Second, publication bias was not assessed, and no attempts were made to identify unpublished studies. Third, details were missing in the studies reviewed. The authors of the studies reviewed responded to emails requesting additional information about their study; however, to facilitate systematic reviews and meta-analyses and to ensure rigor and transparency in research, researchers should adhere to existing guidelines for the conduct and reporting of trials (eg, Consolidated Standards of Reporting Trials, Transparent Reporting of Evaluations with Nonrandomized Designs [44,70]). Fourth, the search strategy used may not have identified all trials published on this topic. In an attempt to minimize this, the reference lists from previously published articles retrieved in the database search were scanned. Finally, adolescent cancer survivors were defined as individuals with cancer in their teenage years (ie, 13-19 years), which is in line with the range used by the Public Health Agency of Canada [27], other researchers [71], and an existing review of symptom clusters in adolescent oncology [12]. As a result, studies containing samples fitting different definitions or those with samples comprised of ≤50% of boys and girls aged 13-19 years were excluded.

On the basis of the current review, there is insufficient evidence available to conclude that PA affects adolescent cancer survivors’ health and QOL. The lack of RCTs and CCTs stands in stark contrast to the extant literature providing evidence for the effects of PA on health and QOL in younger [19,20] and older cancer survivors [21,22]. More high-quality research exploring the effects of PA on health and QOL outcomes in samples containing only adolescent cancer survivors is necessary because PA could offer a cost-effective, non-pharmacological, self-managed strategy to help adolescents manage the burden of cancer. To improve the quality of evidence-based medicine, studies should use RCT or CCT designs, have adequate sample sizes to detect minimal clinically important differences, and ensure intervention dosage is sufficient to elicit changes in the desired outcomes (ie, frequency, intensity, type, duration). Furthermore, studies should also assess the effects of PA on a broad range of biological, physical, psychological, emotional, social, and cognitive health outcomes. Finally, using the Physical Exercise Across the Cancer Experience framework [72] may ensure adolescents at different phases of the cancer trajectory (eg, during treatment, survivorship, palliation) are included in PA trials. This framework could not only help guide researchers seeking to examine the effects of PA across the entire cancer experience but also help answer questions about the optimal time to implement PA interventions for adolescent cancer survivors.

## Appendix

Multimedia Appendix 1 Characteristics of the included physical activity interventions.

Multimedia Appendix 2 Measures and outcome results from the included physical activity interventions.

## Abbreviations

BMC: bone mineral content
BMD: bone mineral density
CCT: controlled clinical trials
PA: physical activity
QOL: quality of life
RCT: randomized controlled trial
