# Supplementary material for: The Effects of Physical Activity on Health and Quality of Life in Adolescent Cancer Survivors: A Systematic Review
Source: JMIR Cancer. 2016 May 24;2(1):e6. doi: 10.2196/cancer.5431 (PMC5369629; doi:10.2196/cancer.5431)
Supplement: Multimedia Appendix 1 [file cancer_v2i1e6_app1.pdf]

Multimedia Appendix 1: Characteristics of the included physical activity interventions.

Study	Study Design and Country	Sample Characteristics	Intervention Characteristics	Theory-Based (Yes/No)	Intention-to-Treat Analysis (Yes/No)
Müller et al [58]	• Controlled clinical trial<br>• Germany	Total Sample<br>• *N* = 21 males and females<br>• M_{age}= 14.0 years ± 2.7<br><br>Type of Cancer Diagnosed<br>• Malignant bone tumor in the lower extremity<br><br>Treatment Status<br>• Recruited after neo-adjuvant treatment, during adjuvant treatment<br><br>Intervention Group (*n* = 10)<br>• M_{age} = 15.5 years ± 2.0 (range 11.5 - 18.1)<br><br>Control Group (*n* = 11)<br>• M_{age} = 12.2 years ± 2.6 (range 8.7 - 17.6)	Supervised<br>• Yes, by 2 trained sports therapists<br><br>Setting<br>• Hospital-based<br><br>Intervention Length<br>• 8-12 inpatient stays (spanning 6 months)<br><br>Frequency and Duration<br>• 5 times/week, participants advised to attend the PA session at least every second day<br>• 15-45 minutes/session<br><br>Intensity<br>• Moderate-to-vigorous<br><br>Activity Type<br>• Stationary bicycling, walking or jogging on the treadmill, or using an elliptical trainer<br>• Strength training (ie, multiple joint exercises such as squats, lunges, rowing)	• No	• No

			• Balance and flexibility training<br>• Sports games (eg, football, basketball, table tennis)		
Rosenhagen et al [59]	• Controlled clinical trial<br>• Germany	Total Sample<br>• N = 20 males and females<br>• M_{age} = 15.3 years ± 3.7<br><br>Type of Cancer Diagnosed<br>• Mixed cancers<br><br>Treatment Status<br>• Recruited during isolation phase of peripheral blood stem cell transplant<br><br>Intervention Group (n = 10)<br>• M_{age} = 14.4 years ± 3.4 (range 10.0 - 20.0)<br><br>Control Group (n = 10)<br>• M_{age} = 13.6 years ± 4.3 (range 6.0 - 19.0; without outlier 11.0 - 19.0)	Supervised<br>• Yes, by trained sports therapists<br><br>Setting<br>• Hospital-based<br><br>Intervention Length<br>• 5-7 weeks<br><br>Frequency and Duration<br>• 3 times/week<br>• 50 minutes/session<br><br>Intensity<br>• Average .6 watt/kg on a stationary bike<br><br>Activity Type<br>• Stationary bicycling<br>• Strength training using barbells, balls, and participants' own bodyweight (eg, squats, side steps, balancing on 1 leg)	• No	• No

M_{age}: mean age.
