# Supplementary material for: The Effects of Physical Activity on Health and Quality of Life in Adolescent Cancer Survivors: A Systematic Review
Source: JMIR Cancer. 2016 May 24;2(1):e6. doi: 10.2196/cancer.5431 (PMC5369629; doi:10.2196/cancer.5431)
Supplement: Multimedia Appendix 2 [file cancer_v2i1e6_app2.pdf]

Multimedia Appendix 2: Measures and outcome results from the included physical activity interventions.

Study	Measures		Outcomes	
	Health and QOL	Other	Health and QOL	Other
Müller et al [58]	Bone Mass<br>• Dual-energy X-ray absorptiometry	Intervention Adherence<br>• Total number of times the intervention was delivered out of the total number of times the intervention was to be delivered<br><br>PA Levels<br>• Accelerometry	Bone Mass<br>• BMC, BMD, and height-corrected lumbar spine Z-scores decreased in both groups over time (*P*-values not reported)<br>• BMC did not differ significantly between groups (*P*s ≥ .35) at any site post-intervention or at follow-up<br>• Lumbar spine BMD differed significantly between groups post-intervention (*P*s = .03), but nonsignificantly at follow-up (*P*s ≥ .29)<br>• Femur and calcaneus BMD did not differ significantly between groups post-intervention or at follow-up (*P*s ≥ .07)<br>• Height-corrected lumbar spine Z-scores differed significantly between groups post-intervention (*P* = .04), but nonsignificantly at follow-up (*P* = .06)	Intervention Adherence<br>• 77% (intervention was received 34.5 times out of 44.8 time it was scheduled to be received)<br><br>PA Levels<br>• At post-intervention (ie, 6 months after baseline), the intervention group engaged in more gait cycles/day, gait cycles/hour, and moderate PA/day (*P*-values not reported; *d*s = 1.44, 1.34, 1.27, respectively) than the control group<br>• At follow-up (ie, 12 months after baseline), the intervention group engaged in more gait cycles/day, gait cycles/hour, and minutes of moderate PA/day (*P*-values not reported; *d*s = 1.64, 1.42, 1.49, respectively) than the control group

				Adverse Event(s)<br>• None reported
Rosenhagen et al [59]	Grip Strength<br>• Hand-held dynamometry<br><br>Fatigue<br>• Participant report<br><br>QOL<br>• Participant report	Intervention Acceptance<br>• Participant report	Grip Strength<br>• Grip strength increased nonsignificantly from baseline to day 14 (*P*-value not reported), but returned to baseline levels post-intervention (*P*-value not reported)<br>• Grip strength scores were not compared between groups<br><br>Fatigue<br>• Fatigue scores improved nonsignificantly over time (*P*-values not reported)<br>• Fatigue scores were not compared between groups<br><br>QOL<br>• General QOL decreased nonsignificantly from baseline to day 14 (*P*-value not reported), but increased nonsignificantly from day 14 to post-intervention, surpassing baseline levels (*P*-value not reported)<br>• Cancer-specific QOL increased nonsignificantly from day 1 to post-intervention (*P*-values not reported)<br>• QOL scores were not compared between groups	Intervention Acceptance<br>• High acceptance for PA intervention in the intervention group, but not in the control group<br><br>Adverse Event(s)<br>• None reported

BMC: bone mineral content; BMD: bone mineral density; *d*s: Cohen's *d* effect sizes; PA: physical activity; QOL: quality of life.
